# *Mycoplasma pneumoniae*–associated Central Nervous System Manifestations: Current Knowledge and Challenges

**DOI:** 10.1093/ofid/ofag333

**Published:** 2026-06-04

**Authors:** Oluwafemi M Akinnurun, Roger Dumke

**Affiliations:** TU Dresden, Institute of Medical Microbiology and Virology, Dresden, Germany; TU Dresden, Institute of Medical Microbiology and Virology, Dresden, Germany

**Keywords:** CNS complications, diagnosis, extrapulmonary manifestations, *Mycoplasma pneumoniae*, treatment

## Abstract

**Background:**

*Mycoplasma pneumoniae* is a common cause of human respiratory infections. Extrapulmonary manifestations include diseases of the central nervous system (CNS).

**Methods:**

To summarize the current knowledge on *M. pneumoniae*–associated CNS complications, cases published in PubMed between 2015 and 2025 were reviewed.

**Results:**

We included 70 studies with 207 patients. Various neurological manifestations were identified, affecting patients between the ages of 3 and 69. Cases of encephalitis predominated (72%). Diagnosis of *M. pneumoniae* involvement was confirmed primarily by the detection of specific antibodies in serum. The prodromal phase between respiratory and neurological symptoms (<7 vs ≥ 7 days) was distributed almost evenly. In most cases, antibiotics effective against mycoplasma are used (88%), and in 81% of patients, complete recovery was achieved.

**Conclusions:**

While the administration of antibiotics is accepted for the treatment of CNS manifestations, the use of other therapies is guided by the severity of the course and empirical considerations.

## Teaser

Respiratory infections caused by *Mycoplasma pneumoniae* can be associated with diseases of the central nervous system. After evaluating the publications on CNS manifestations (2015-2025), the diagnostic procedures used, the therapies employed, and the outcome of the cases are summarized.

*Mycoplasma pneumoniae* is a representative of the Mollicutes group, which is characterized by significant genome reduction and limited metabolic capabilities. The most striking expression of this genome reduction is the absence of a classic bacterial cell wall. Among the species relevant to humans, *M. pneumoniae* is a common cause of community-acquired respiratory infections, affecting all age groups, but predominantly children and young adults. The pathogen is transmitted by aerosols after close human-to-human contact. The course of the disease is characterized by relatively long incubation periods of up to 3 weeks, after which an uncomplicated tracheobronchitis develops in most cases [1]. These mild cases are at least 20 times more common than pneumonia, and up to 20% of infections are asymptomatic [2]. Reported infection clusters are concentrated in families, schools, daycare centers, and military camps [1]. On a global scale, outbreaks emerge periodically, roughly every 3–7 years [1]. An interesting trend in the infection rates was demonstrated during and after the COVID-19 pandemic. The introduction of strict hygiene measures to reduce the transmission of COVID-19 infections also led to extremely low incidences of *M. pneumoniae* worldwide [3]. After these measures were lifted, high clusters of infections were reported in many countries in 2023–2024 [4]. This fluctuation is thought to be due to a decrease in specific immunity in the period before the increase in incidence and/or a change in the genotypes predominantly circulating in the population, which differ in their antigenic surface proteins at the amino acid level (eg, in the main P1 adhesin) [5].

Although *M. pneumoniae* respiratory infections are often mild and self-limiting, many patients do experience severe diseases that require hospitalization [6–9]. Antibiotic treatment is challenging because the bacteria are intrinsically resistant to beta-lactam antibiotics due to the absence of a cell wall. Other effective therapeutic options (fluoroquinolones and tetracyclines) can only be used to a limited extent in the pediatric age group due to the side effects. Owing to their efficacy and tolerability, macrolides are therefore favored and predominantly prescribed. Despite the fact that the respiratory tract is the entry point for the bacteria into the host organism and the primary site of infections by *M. pneumoniae*, diverse extrapulmonary manifestations have also been described. These include involvement of the skin, the cardiovascular, digestive, urogenital tract, and the hematological system [10]. In addition, various *M. pneumoniae*–associated neurological manifestations can affect both the peripheral and central nervous system (CNS) [11]. Extrapulmonary complications can affect up to 30% of people with respiratory infections [12–14].

In this review, we have summarized the recent findings on the incidence, clinical features, diagnosis, treatment, pathogenesis, and outcome of CNS manifestations of *M. pneumoniae* from an infectious disease perspective. In various studies conducted in recent years, the diagnostic value of the tests used in these reports has been assessed differently. In addition, relevant diagnostic methods are usually initiated to varying extents in clinical practice. Given the inconsistency in treatment regimens observed in the present study, this information gap evidently also applies to the selection of optimal therapeutic steps for these conditions. Therefore, the aim of the present review is to contribute to an updated approach to the diagnosis and treatment of these often-severe complications of the frequent respiratory infections caused by *M. pneumoniae*.

## METHODS

English-language publications (full texts) listed in PubMed between January 2015 and October 2025 (searches for: “Mycoplasma pneumoniae and encephalitis/CNS/encephalopathy/meningoencephalitis/meningitis/stroke/myelitis/cerebellitis/optic neuritis”) were evaluated. This period was chosen because earlier reviews on different aspects of the topic are available [10–12, 15–17]. Inclusion was based on the studies' content, regardless of their geographic location. The abstracts of the identified studies were evaluated by both authors. The following information was ideally included in the review: year of publication, definition of CNS manifestation, age and sex of the patient, mention of causes of immunosuppression, diagnostic test(s) to confirm *M. pneumoniae* involvement, timing of the prodromal stage, treatment administered, and outcome. These criteria also applied to cases in case series in which, in addition to *M. pneumoniae*, other infectious causes of the patients' neurological diseases were listed. Studies without definition of the infectious causes of the neurological manifestation were excluded. Seven articles were not considered due to missing data on the patient, the diagnostic procedures performed, and the therapy. In contrast, studies with a clear link between CNS manifestation and *M. pneumoniae* infection were included even if certain details were not mentioned (gender, immunosuppression, details of antibiotic therapy, prodromal stage, or outcome). These cases appear as “not specified” in the analyses. A total of 70 studies were included in the review.

### INCIDENCE AND PATHOGENESIS

Central nervous system diseases are a frequently reported extrapulmonary manifestation of *M. pneumoniae* infections [18, 19]. The first description of a CNS complication of infection dates back to 1956 [20]. It is estimated that these neurological manifestations arise in ∼0.1% of all *M. pneumoniae* infections [1]. However, current studies have found rates of up to 3% [13, 14, 21]. In recent decades, it has become apparent that these diseases often constitute medical emergencies. However, relatively mild courses of CNS complications have also been reported. The disease spectrum is quite diverse ranging from encephalitis, meningoencephalitis, optic neuritis to transverse myelitis and others (Table 1) [10, 11, 22]. The most frequently published and best studied CNS manifestation is the *M*. *pneumoniae*–associated encephalitis, but it can be caused by many pathogens [25]. The term *M. pneumoniae*–associated encephalitis is used primarily to describe cases of encephalitis that are suspected to be associated with a concurrent or prior *M. pneumoniae* infection. However, a registered history of prior or concurrent respiratory tract infection is not always obligatory for the development of CNS complications [1, 26]. Studies demonstrated that only an estimated 52%–77% of patients with CNS manifestation due to *M. pneumoniae* had preceding or concurrent respiratory infections [27]. Furthermore, *M. pneumoniae* encephalitis is quite difficult to describe, as many published cases would not meet all the criteria for an exact definition of disease causation [25]. Mainly investigated among children, *M. pneumoniae*–associated encephalitis is thought to represent up to 13% of encephalitis cases [1, 10, 11, 13, 28–30] and has been found to be the major etiology of acute pediatric encephalitis [1, 31]. Among confirmed *M. pneumoniae* infections, the rate of encephalitis cases is described as <1% [18, 19, 26]. However, a recent study from France estimates the rate of meningoencephalitis cases among hospitalized adults at 1.5% [32]. Deaths from this condition have been reported [27, 28], but the overall mortality rate is low.

**Table 1.** Central Nervous System Manifestations of *M. pneumoniae* Infections [10, 11, 22, 23] Considered in the Present Review (2015–2025, 70 Studies With 207 Cases)

Manifestation	Number of Cases	Age Range of Patients (y)^a^	Mean Age of Cases (y)^a^
Encephalitis/meningoencephalitis	148	3–61	13.2
MERS	11	8–40	19.6
Ischemic stroke/infarction	11	5–43	16.5
Cerebellitis/cerebellar ataxia	8	4–26	10.0
ADEM	8	4–38	17.4
Optic neuritis	7	7–69	31.0
Transverse myelitis	6	4–65	27.5
Aseptic meningitis	5	4–21	11.2
Striatal necrosis, vasculitis, rhabdomyolysis	1 each	9, 32, 28	…

Abbreviations: ADEM, acute disseminated encephalomyelitis; MERS, mild encephalopathy with reversible spinal lesion.

^a^Without study of Fan et al [24] (n = 87, only mean age available).

Despite relatively frequent occurrence, the pathogenesis of these CNS complications is still not fully clear. Various postulations about the disease mechanism include: a direct bacteria invasion of the CNS with presence of bacteria at the site of inflammation and local induction of cytokines such as interleukin (IL)-18, IL-8, Th-1, and Th-2 [33], or indirectly as a result of immune-mediated damage such as autoimmunity, allergy or immune complex production, or a hypercoagulable state leading to intravascular coagulation and thromboembolic phenomenon of the CNS [10, 11, 19, 28]. Two different clinical patterns of *M. pneumoniae*–associated encephalitis have been described [10, 19, 34] representing the pathogenesis mediated directly by the bacteria or by autoimmune processes: a first pattern with a shorter prodrome (<7 days) before the onset of neurologic symptoms (early-onset) and a second pattern with a prolonged prodrome (≥7 days) before the onset of neurological symptoms (late-onset). Certain autoantibodies have been identified in some patients with *M. pneumoniae* infections that later develop neurological complications. It is thought that *M. pneumoniae* antigens act as molecular mimicry to components of myelin, leading to the development of intrathecal autoantibodies [34]. These antibodies include antigalactocerebroside (GalC) antibody [35], myelin oligodendrocyte glycoprotein antibody [36], anti-IgLON5 antibody [37], anti-N-methyl-D-aspartate receptor antibody [38], anti-GM2 [39], and anti-GQ1b antibody [40]. It has been shown that antibodies against *M. pneumoniae* react with GalC in patients with Guillain–Barré syndrome [41]. The identification of autoimmune antibodies in many cases of *M. pneumoniae*–associated CNS diseases further supports the hypothesis of the involvement of an autoimmune-mediated process in the pathogenesis [23]. It is however important to note that all these factors may not be mutually exclusive and that multiple mechanisms may be occurring within a patient [19, 31, 42, 43].

## RESULTS OF LITERATURE REVIEW

**Figure 1.**
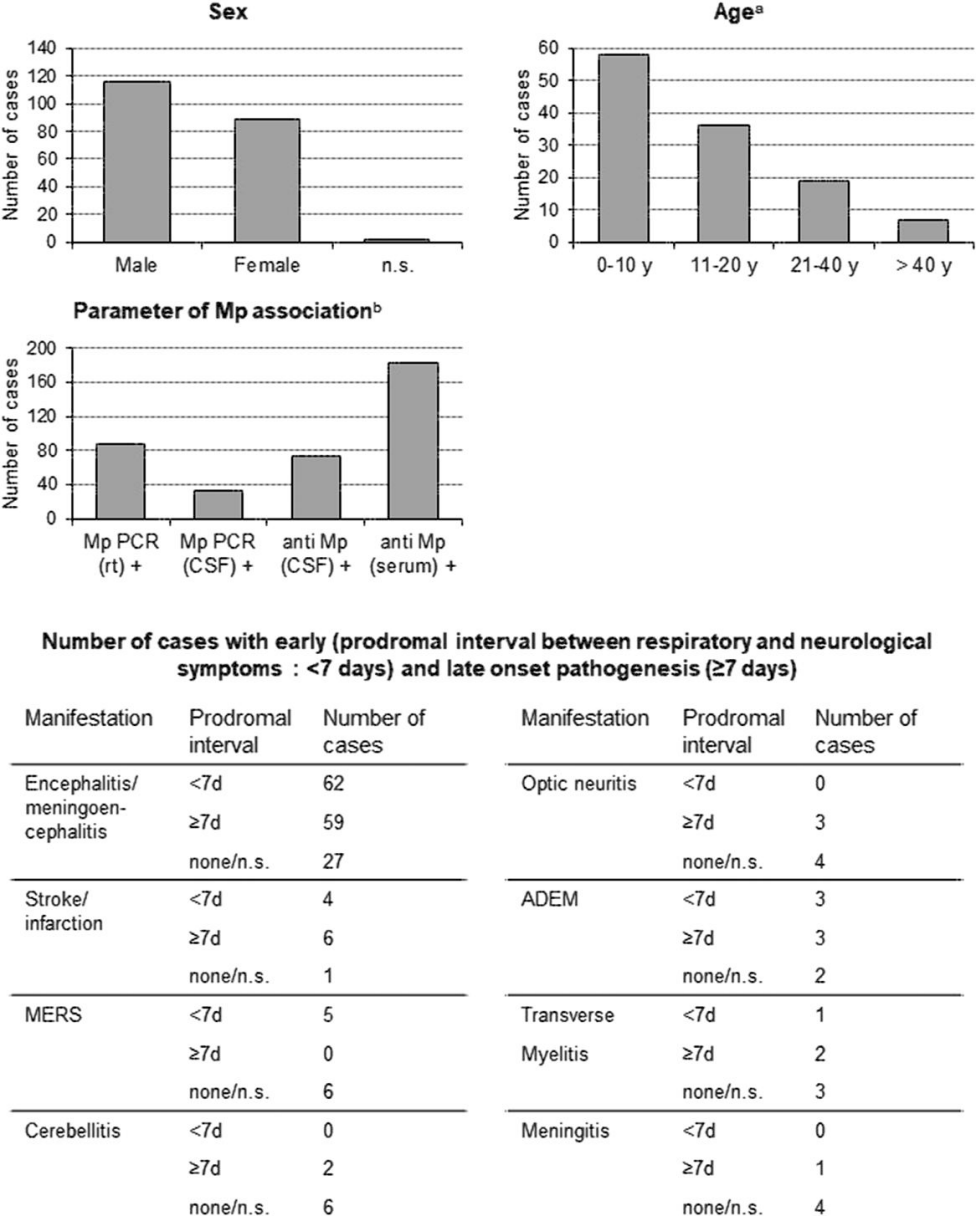
Characteristics of cases of *M. pneumoniae*–associated CNS manifestations published 2015–2025 (70 studies with 207 cases). +, positive; ADEM, acute disseminated encephalomyelitis; CSF, cerebrospinal fluid; Mp, *M. pneumoniae*; MERS, mild encephalopathy with reversible spinal lesion; n.s., not specified; rt, respiratory tract. ^a^Without study of Fan et al [24] (n = 87, only mean age available). ^b^Multiple responses possible.

Between January 2015 and October 2025, 70 studies involving 207 patients were listed in PubMed on CNS manifestations of *M. pneumoniae* infections. Of the included studies, the majority (80%) were case reports, and 20% were case series involving 2–87 patients (4 of which were retrospective). The cases predominantly affected males (56.6%, Figure 1). The clinical features of the CNS diseases can be classified into 9 manifestations (Table 1), with encephalitis dominating at 71.5%. The age of the affected patients ranged from 3 to 69 years, with 0–10 (48.3%) and 11–20 years (30.0%) being the predominant groups. Interestingly, a preexisting condition that could be associated with immunosuppression was reported in only 4 out of 207 cases (diabetes, asthma, multiple sclerosis, and Hashimoto's disease). To confirm an association between CNS symptoms and *M. pneumoniae*, the determination of specific IgM or IgG antibodies in serum was predominantly used (88.4%), followed by PCR detection in the respiratory tract (42.5%) and antibody detection in cerebrospinal fluid (CSF, 35.7%). *Mycoplasma pneumoniae*–specific PCR from CSF was positive in only 15.5% of patients. If reported, the duration of the prodromal interval between respiratory and neurological symptoms (<7 vs ≥7 days), which is important for assessing the pathogenesis, is almost equally distributed in cases of encephalitis (62 vs 59 cases, Figure 1). In contrast, patients with other CNS manifestations except encephalitis (relatively small number of cases) are predominantly found in the interval <7 days (mild encephalopathy with reversible spinal lesion [MERS]) or ≥7 days (optic neuritis). Pleocytosis or elevated protein concentration was found in the CSF of 58.5% and 49.7% of patients and, therefore, cannot be considered a reliable clinical parameter for the CNS complication of an infection. A broad spectrum of autoantibodies was examined in 58 patients and tested positive in 39.7% of cases.

**Figure 2.**
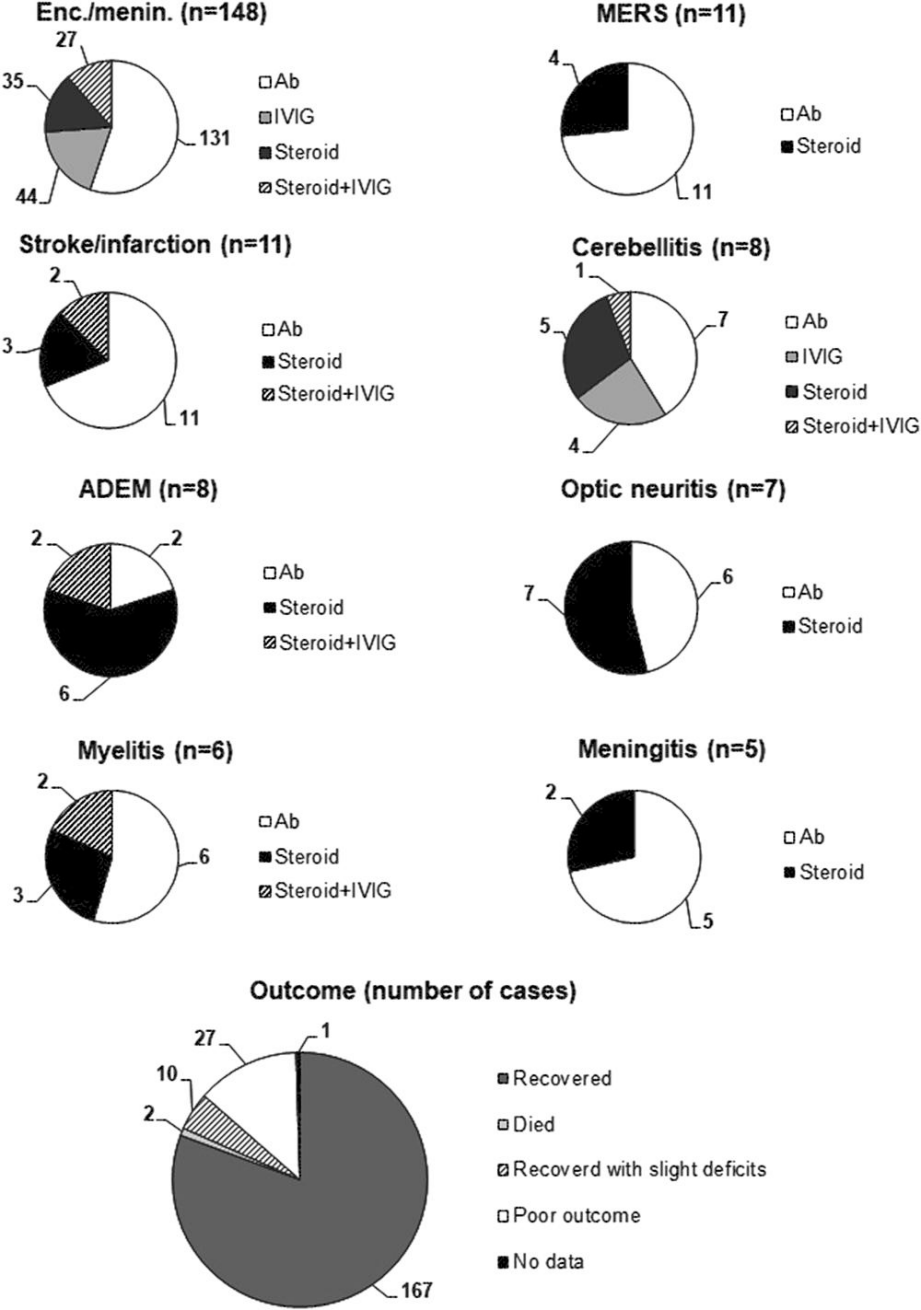
Treatment and outcome of cases of *M. pneumoniae*-associated CNS manifestations. ADEM, acute disseminated encephalomyelitis; ab, antibiotics effective to mycoplasmas; Enc./menin., encephalitis/meningoencephalitis; IVIG, intravenous immunoglobulin; MERS, mild encephalopathy with reversible spinal lesion.

When neurological symptoms occurred and there were indications of *M. pneumoniae* involvement, 87.9% of cases were treated with antibiotics that are effective against mycoplasma (Figure 2; Supplementary Table 1). Combinations (macrolides, tetracyclines, and/or fluoroquinolones) were used in 6 patients. Macrolides alone were applied to treat 166 cases (80.2%), with azithromycin being the most commonly used (76.5%). Clarithromycin (7.2%) and erythromycin (2.4%) were administered considerably less frequently. In 13.9% of cases, the macrolide was not specified. Tetracyclines (doxycycline and minocycline) or fluoroquinolones (ciprofloxacin, moxifloxacin, and levofloxacin) were used exclusively in 21 patients. Importantly, these antibiotics were also applied to successfully treat 9 children between the ages of 4 and 10, with no side effects reported. Although many studies lacked data on dosage and duration of administration, the dosage of antibiotics varied considerably in the remaining cases. Only macrolides were administered at a largely uniform dose of 500 mg/day (children: 10 mg/kg/day). The duration of treatment ranged from 3 to 15 days (for macrolides: mostly 7–10 days). Particularly in countries with high rates of macrolide resistance (eg, China), treatment was switched to tetracyclines and fluoroquinolones when macrolides failed to produce therapeutic success. Immunomodulatory therapies were used in 150 patients (72.5%): intravenous administration of immunoglobulins (IVIGs, 23.2%), steroids (32.4%), and IVIG + steroids (16.9%). Plasmapheresis was performed exclusively in combination with steroids or steroids + IVIG (4 cases each, Supplementary Table 1). One study confirms the significantly shorter duration of clinical improvement and of hospitalization when macrolides are used in combination with IVIG or steroids [24].

The outcome of CNS manifestations involving *M. pneumoniae* can be considered predominantly favorable (Figure 2). In 81.1% of cases, complete recovery was achieved after varying lengths of treatment. However, mild deficits after completion of treatment (eg, vision problems, autonomic dysfunction, minimal hand weakness) were observed in 4.8% of patients, and a poor outcome (different and severe neurological dysfunctions, low Glasgow coma scale, and loss of vision) was diagnosed in 13.1% of patients. Two individuals with severe preexisting conditions (aged 37 and 58, respectively) died from CNS complications (ischemic stroke and hemorrhagic leukoencephalitis [25, 33], in Supplementary Table 1).

## CURRENT CHALLENGES

### Clinical Aspects and Therapy of Neurological Manifestations of *M. pneumoniae* Infections

The respiratory symptoms induced by *M. pneumoniae* are not clearly distinguishable from those of other bacteria and viruses that can cause community-acquired infections. Coinfections are detected relatively frequently worldwide [eg, 45–47], and asymptomatic carriers of the bacteria have been confirmed [48]. In addition, there are mild and inconspicuous courses of infections, some of which may occur well before the onset of neurological manifestations. These facts underscore the differential diagnostic challenges for clinical practice (see below). The current situation could be the reason why the treatment of *M. pneumoniae*–associated CNS manifestations is not based on a uniform therapy regimen. On the other hand, infections caused by mycoplasma may require targeted therapeutic intervention. This explains why antibiotics effective against mycoplasma were used in the majority of cases of suspected *M. pneumoniae*–associated CNS manifestations. The aim is to curb ongoing acute inflammation by eradicating the triggering antigen [11]. However, several studies reported that effective antibiotics were only used after the diagnostic evidence of a *M. pneumoniae* involvement was available, which in turn can lead to delays in the elimination of the bacteria. This is, however, contradicted by the fact that cases with suspected involvement of *M. pneumoniae* in which antibiotics were not used (9.7%, Supplementary Table 1) do not appear to take a more severe course. In addition, treatment failures due to a lack of penetration of the therapeutic agents into the CNS [49] or an autoimmune-mediated manifestation without presence of *M. pneumoniae* in the CSF should be mentioned here. Despite these limitations, clinicians should be made aware of the need to include *M. pneumoniae* in the differential diagnosis of infection-related CNS symptoms, to initiate appropriate diagnostics and, if necessary, adequate antibiotic therapy. Several studies also emphasize the importance of early antibiotic treatment for the outcome of these CNS manifestations [28, 43, 50, 51]. It should be noted that the macrolides predominantly used for this purpose are limited in their effectiveness in certain regions (mainly East Asia) due to frequent mutations of the 23S rRNA of *M. pneumoniae*. This aspect plays a role in only a few studies [52–54] but may also become relevant in countries with relatively low rates of macrolide resistance due to factors such as migration, visits, travels, and emergence of resistance during therapy. A recent meta-analysis confirmed an increased incidence of extrapulmonary complications after infection with resistant strains compared with sensitive strains [44]. If diagnostic options are available, failure to respond to antibiotic therapy should prompt molecular testing for mutations of 23S rRNA and, if necessary, a rapid change of antibiotic. Although the importance of antibiotics in CNS complications of *M. pneumoniae* infections seems to be widely accepted [55], there are no case–control studies available that prove the benefit of antibiotic treatment for the outcome of neurological manifestations. This also applies to the use of anti-inflammatory therapies (steroids and IVIGs). An earlier literature review found successful treatment rates of 17% (IVIG) and 28% for cases of autoimmune-mediated encephalitis [56]. The necessary therapies are also obviously dependent on the severity of the CNS manifestation [57]. However, some authors emphasize the early use of immunomodulatory treatments to minimize neuronal damage [43, 58], and initial evidence shows shortening of the disease duration with steroids in severe respiratory infections caused by *M. pneumoniae* [59, 60]. Future studies should help to clarify which clinical parameters can be used as criteria for a decision to apply such therapeutic interventions.

### Diagnosis of Neurological Manifestations of *M. pneumoniae* Infections

Assessing the validity of the serological and molecular methods that can be used to confirm the involvement of *M. pneumoniae* in neurological complications is difficult. For the reasons mentioned in Table 2, molecular detection of *M. pneumoniae* from the respiratory tract and/or detection of specific antibodies in serum is indicative of the pathogen's involvement in CNS complications but is not definitive proof. In principle, PCR detection of *M. pneumoniae* in CSF can be regarded as evidence of the pathogen's involvement in a case of CNS manifestation. However, its detection was only successful in a small number of patients (32/206, 15.5%, Supplementary Table 1). Due to the low PCR detection rates of *M. pneumoniae* in CSF reported in these studies, it is probable that a significant proportion of cases are postinfectious or immune-mediated in nature [61]. On the other hand, early detection of the bacteria in the CNS without an increase in serum IgM and/or PCR detection in the respiratory tract cannot be ruled out [55]. Presence of specific *M. pneumoniae* antibodies in CSF is also an indication of the role of *M. pneumoniae* in the inflammatory process (here, 35.7% of cases). Compared with the other studies considered in this review (positive PCR/antibody detection in CSF: 5.9%/3.4%), both overall mean rates are significantly elevated by the large number of patients (n = 87) included in 1 study (corresponding rates: 28.7%/80.4% [24]). Evaluation of the integrity of the blood–brain barrier with the quantitative analysis of serum/CSF pairs increases the informational value of serological diagnostics [62, 63]. The disadvantage of this approach is the effort required to perform the quantitative comparison (parallel determination of albumin and total immunoglobulin in serum and CSF). Furthermore, testing for specific DNA and/or antibodies in CSF has its challenges. The commercially available kits for detecting corresponding antibodies in serum or DNA in respiratory samples have not been validated for use in CSF. Although one might expect that the detection of anti-*M. pneumoniae* immunoglobulins and specific DNA in CSF would be possible with these tests, the regulatory obligations involved pose a problem for the quality management of a diagnostic laboratory and, thus, limiting the ability to offer such tests. For practical reasons, the studies included in this review often detect specific antibodies in serum (mostly IgM) as the sole indication of *M. pneumoniae* involvement (38.2% of cases, Supplementary Table 1). This approach is known from studies in the past [31, 34, 64] and understandable from a clinical perspective, but should then include the careful exclusion of other causes [36]. The classification of diagnostic results into possible (single elevated specific antibodies in serum and/or PCR detection in the respiratory tract), probable (4-fold specific antibody increase in serum), or confirmed involvement (*M. pneumoniae* detection in CSF or specific intrathecal antibody response) of this pathogen in CNS symptoms, as used with slight variations in many reports, should still be considered valid [25, 26]. The diagnosis “confirmed *M. pneumoniae* involvement” could only be made in a few cases in the studies included [37, 53, 59, 62]. The combination of serological/molecular methods for investigation of respiratory samples, serum, and CSF is considered optimal for diagnosis [29] but is often not feasible for practical reasons. However, this evidence should be aimed for in future reports.

**Table 2.** Evaluation of Diagnostic Procedures for Confirmation of an Involvement of *M. pneumoniae* (Mp) in Cases of CNS Symptoms

Procedure	Advantages	Disadvantages
PCR (rt)	−High specificity/sensitivity −Many validated kits available	−Negativity in late stages of infection as well long-term positivity after acute phase of infection possible −Positive in asymptomatic carriers of infection −Results depend on investigation of suitable specimens
PCR (CSF)	−If positive, clear indication for an involvement of Mp −High specificity/sensitivity can be expected −CSF in most cases available	−Available kits not validated for CSF −Negative in late stages of infection −Negative in autoimmune-mediated cases
Serodiagnosis (serum)	−Simple sampling −Use of specific A, M, and/or G immunoglobulins for detection	−Specificity/sensitivity depends on the kit used −Negative in early stages of infection (≤7 d) −Positive in asymptomatic carriers of infection −IgM might be negative after re-infection of adults −Long-term presence of antibodies after acute infection −Availability of paired sera (IgG)
Serodiagnosis (CSF)	−Use of specific A, M, and G immunoglobulins for detection −CSF in most cases available	−Available kits not validated for CSF −Results depend on the integrity of blood–brain barrier −Negative in autoimmune-mediated cases
Antibody index (serum/CSF)	−Confirmation of an intrathecal production of specific antibodies	−Nearly simultaneous sampling of serum/CSF pairs −Further parameter (serum albumin and whole immunoglobulins) must be available −Kits not validated for CSF −Specificity/sensitivity depends on the kit used

Abbreviations: CSF, cerebrospinal fluid; Mp, *Mycoplasma pneumoniae*; rt, respiratory tract.

### Epidemiological Aspects

Central nervous system complications of *M. pneumoniae* infections show a broad spectrum of clinical presentations, which are dominated by encephalitis in terms of numbers but can also include other manifestations (Table 1). The differentiation may require extensive neurological expertise, which is not the subject of this review. Although largely unclear, different pathomechanisms, the influence of coinfections, strain characteristics, and individual factors (eg, the patient's immune status) can be assumed to be causes of these differences in type of neurological symptoms presented. Among the studies considered, this results in different therapeutic approaches, which are obviously guided by the severity of the disease course and empirical considerations. Typing of strains has been an aspect of epidemiological research for years, especially in respiratory infections caused by *M. pneumoniae* [65]. The antigenically different P1 types are of particular interest with regard to the influence on the host's immune response [66]. However, there are only a few studies on extrapulmonary manifestations [67, 68], which makes it difficult to estimate these effects, and corresponding studies on CNS complications are missing. Therefore, the influence of subtypes that change in their quantitative distribution over different periods of time [69] on the development and course of neurological manifestations of the infection can currently neither be confirmed nor ruled out.

Several authors pointed out that the above-mentioned increases in the incidence of respiratory infections caused by *M. pneumoniae* were the reason for targeted investigations of extrapulmonary manifestations [62, 70, 71]. Applying this logic, one would have expected cases involving the CNS to increase during these periods as well. Interestingly, recent reports of a noticeable increase in such cases are rare [32], despite the exceptionally high number of *M. pneumoniae* infections in many regions of the world in 2023/2024. However, due to the diagnostic challenges mentioned above and the often-mild course of the disease, this could also be due to these CNS manifestations being underdetected.

## LIMITATIONS

This review has limitations. The study included only those aspects of CNS manifestations of *M*. *pneumoniae* infections that have been published in PubMed. It can be assumed that there is a high number of unreported cases due to their lack of publication. The often-mild clinical course and the large number of previously described cases, for example, involving encephalitis, support this assumption. This fact could also lead to more serious or complex cases being published. Quantitative comparisons between the various forms of CNS complications or figures on the current prevalence of neurological manifestations are only possible in long-term and detailed case–control studies. Furthermore, errors in the identification of additional studies during the period under review cannot be ruled out. However, the selection of search terms was broad, so this should affect only a few isolated cases. Furthermore, we have noted that the incidence of *M*. *pneumoniae* infections has decreased drastically during the COVID-19 pandemic. It can be assumed that this has also reduced the number of neurological manifestations of the infection compared with nonpandemic periods and could lead to fewer cases being reported in the scientific literature. Quantitative data on this aspect cannot be provided. However, since there is no consistent evidence of a considerably higher rate of extrapulmonary complications from infections following the pandemic, the conclusions regarding the pathogenesis, diagnosis, and treatment of CNS manifestations should remain largely unaffected.

## CONCLUSION

Central nervous system manifestations of *M. pneumoniae* infections remain a diagnostic and therapeutic challenge in the field of infectious diseases. Besides other clinical pictures, encephalitis is the most common of these CNS complications and is associated with a risk of severe neurological sequela. A combination of different pathomechanisms, the presence of coinfections, strain characteristics, and the patient's immune status are some of the possible factors that may contribute to the diverse disease spectrum and severity of *M. pneumoniae*–associated CNS diseases. In addition, a comprehensive diagnostic approach might be necessary to detect the disease etiology, and the validity of the results of these methods must be carefully evaluated. In addition to the known limitations of the serological and molecular methods currently in use, supplementary validation of these tests can offer benefits for clinical practice. For example, the results of a serum/CSF antibody index based on approved test systems will provide clinicians with additional information. Finally, an early and targeted therapy can lead to favorable clinical outcomes in most patients. This treatment should include antibiotics effective to mycoplasmas and, depending on the clinical course, immunomodulators. However, the review has unfortunately confirmed the continuing uncertainty regarding the practical efficacy of antimicrobial and immunomodulatory treatments as well as the clinical indications for their use, so that further studies are necessary to establish standardized guidelines for therapy.

## Supplementary Material

ofag333_Supplementary_Data

## Supplementary Data

Supplementary materials are available at *Open Forum Infectious Diseases* online. Consisting of data provided by the authors to benefit the reader, the posted materials are not copyedited and are the sole responsibility of the authors, so questions or comments should be addressed to the corresponding author.
