# Supplementary material for: Mycoplasma pneumoniae–associated Central Nervous System Manifestations: Current Knowledge and Challenges
Source: Open Forum Infect Dis. 2026 Jun 4;13(6):ofag333. doi: 10.1093/ofid/ofag333 (PMC13251344; doi:10.1093/ofid/ofag333)
Supplement: ofag333_Supplementary_Data [file ofag333_supplementary_data.pdf]

Table S1. Summary of cases of CNS manifestations associated with *M. pneumoniae* infection published between January 2015 and October 2025 in PubMed.

Age (y)/Sex	Main clinical presentation	Parameter for Mp involvement	Therapy[a]	Diagnosis	Outcome	Ref.
26/m	Fever, cough, weakness	Serum IgM pos.	Azi, IVIG	Cerebellitis	Largely recovered	[1]
60/m	Cough, pneumonia, different neurological problems	Serum IgM pos.	Moxifloxacin, IVIG, steroid	Transverse myelitis	Largely recovered (slight vision problems)	[2]
10/f	Weakness upper limb	Serum IgM pos.	Heparin, azi	Ischemic stroke	Recovered	[3]
37/m	Seizure, confusion	Serum IgM/IgG pos.	Azi, IVIG, plasmapheresis, steroid	Encephalitis	Recovered	[4]
8/?	Respiratory symptoms, dizziness, headache, reduced limb movement	Serum IgM pos., CSF: anti Mp antibodies pos.	Antibiotics (not specified), steroid, IVIG	Encephalitis	Recovered	[5]
9/f	Respiratory symptoms, gait and speech problems	Serum IgM/IgG pos.	Azi, doxycyclin, IVIG	Cerebellar ataxia	Recovered	[6]
21/f	Fever, headache	CSF: Mp PCR pos.	Clarithromycin, doxycycline, levofloxacin	Meningitis, intracranial abscess	Recovered	[7]

9/m, 5/f, 6/f	Cough, fever, headache (2x), seizure (2x) vomiting (2x), hypersomnia (1x), orbital pain (1x)	Mp PCR nasopharynx and serum IgM/ pos.	Erythromycin, steroid	Encephalitis	Recovered	[8]
16/f	Fever, cough, multifaceted neurological problems	CSF: Mp PCR pos., serum IgM/IgG pos., BAL: Mp PCR pos.	Azi, IVIG, plasmapheresis, steroid	Encephalitis	Recovered	[9]
16/m	Fever, respiratory symptoms, multifaceted neurological problems	Serum IgM/IgG pos., tracheal aspirate: Mp PCR pos.	Levofloxacin, steroid, IVIG	Encephalitis	Recovered	[10]
6/m, 6/m	Fever, cough, different neurological and urogenital dysfunctions	Serum IgM pos., one patient: Mp PCR pos. (throat)	Steroid, IVIG (both patients), azi and levofloxacin in one patient	Encephalitis, polyradiculitis	Recovered and recovered with slight autonomic dysfunction	[11]
4/f	Numbness of extremities	Serum IgM/IgG pos.	Azi, steroid	Transverse myelitis	Recovered	[12]
16/f	Fever, cough, neurological and pulmonary problems	Serum IgM/IgG pos.	Azi, doxycycline	Cerebral artery occlusion	Different dysfunctions	[13]

1-14, 56.3% m (87 patients)	Fever (96.5%), respiratory symptoms (94.3%), gastrointestinal, cardial and skin manifestations	Serum IgM pos. (83.9%), CSF: IgM pos (80.5%), CSF: Mp PCR pos. (28.7%), throat: Mp PCR pos (66.7%)	Azi (100%), IVIG (42.5%), steroid (13.8%), IVIG and steroid (6.9%)	Encephalitis	Poor outcome in 17.2% of cases	[14]
38/f	Decreased vision and ocular pain	Serum IgM/IgG pos.	Fluoroquinolone, steroid	Optic neuritis	Recovered	[15]
32/m	Fever, cough, vision problems	Serum IgM pos., nasopharynx: Mp PCR pos.	Azi, steroid	Optic neuritis	Recovered	[16]
69/f	Fever, rash, arthralgia, diplopia	PA pos.	Minocycline, steroid	Optic neuritis, erythema, polyarthritis	Recovered	[17]
5/m	Fever, cough, headache, vomiting	Serum IgM pos.	Azi, steroid, IVIG	Encephalomyelitis	Recovered	[18]
8/f	Fever, cough, headache	Serum IgM pos.	Erythromycin, IVIG, steroid	Meningoencephalitis	Recovered	[19]
25/m	Fever, headache, convulsions	Serum IgM pos.	IVIG, steroid	Meningoencephalitis	Largely recovered	[20]
43/m	Fever, cough, rash, confusion	Serum IgM pos.	Azi	Ischemic stroke	Recovered	[21]
16/f	Blurry vision and ocular pain	Serum Ig pos.	Steroid, plasmapheresis	ADEM	Largely recovered	[22]

58/m	Respiratory symptoms, fever, altered consciousness	Serum IgG pos.	Moxifloxacin, steroid	Hemorrhagic leucencephalitis	Died	[23]
11/f	Fever, different neurological symptoms	Serum IgM pos.	Azi, steroid	Encephalitis	Recovered	[24]
40/f	Different neurological dysfunctions	Mp infection (not specified)	IVIG, steroid	Encephalitis	Recovered	[25]
15/m	Fever, cough, different neurological problems	Serum IgM/IgG pos.	IVIG, steroid	Encephalitis	Recovered	[26]
5/m	Fever, vomiting, diarrhea, weakness	Serum IgM/IgG pos.	Steroid	Encephalitis	Recovered	[27]
38/f	Pneumonia, different neurological dysfunctions	Serum IgM/IgG pos.	Steroid, IVIG, plasmapheresis	ADEM	Recovered	[28]
43/f	Fever, altered mental status	Serum IgM/IgG pos.	Clarithromycin, steroid, IVIG	Encephalitis	Recovered	[29]
5/m	Fever, cough, pneumonia	Serum IgM pos.	Azi, thrombolytic therapy	Ischemic stroke	Largely recovered	[30]
37/m	Fever, headache, speech problems	Serum IgM pos.	Azi	Ischemic stroke, myocarditis	Died	[31]
24/m	Cough, myocarditis, stroke	Serum IgM pos.	Azi	MERS	Recovered	[32]
14/m	Headache, diarrhea, different neurological symptoms	Serum IgM pos.	Azi, steroid	MERS	Recovered	[33]

5/f	Fever, cough, neurological symptoms	Serum IgM pos., respiratory samples and CSF: Mp PCR pos.	Minocycline, steroid	Cerebral infarction	Recovered	[34]
32/m	Cough, different neurological symptoms	Serum IgM/IgG pos.	Azi and doxycycline, steroid, IVIG	Rhabdomyolysis	Recovered	[35]
15/m	Extremity weakness, paresthesia	Serum IgM/IgG pos.	Azi, steroid, plasmapheresis, IVIG	Transverse myelitis	Neurological problems	[36]
4/f	Cough, different neurological symptoms	Serum IgM pos.	Azi, steroid, IVIG	ADEM	Recovered	[37]
28/m	Respiratory symptoms, fever, headache, confusion	Oropharynx: Mp PCR pos.	Macrolide, steroid	CNS vasculitis	Recovered	[38]
17/m	Cough, altered speech and communication	Respiratory sample: Mp PCR pos., PA pos.	Levofloxacin, steroid	MERS	Recovered	[39]
13/m	Fever, cough, abnormal speech, hallucinations	Serum IgM pos.	Clarithromycin, steroid	Encephalitis	Recovered	[40]
14/f, 12/f	Ophthalmoplegia, headache, loss of vision	Serum IgM/IgG pos.	Steroid in both cases	ADEM	Recovered	[41]

7/?	Fever, cough, headache, different neurological problems	Serum IgM pos., pharynx: Mp PCR pos.	Steroid	Encephalitis	Recovered	[42]
9/f	Headache, fever, vomiting, ataxia	Serum IgM pos.	Clarithromycin, steroid	Cerebellitis	Recovered	[43]
22/f	Head and neck pain, nuchal stiffness	Serum IgM pos.	Steroid	ADEM	Recovered	[44]
3/m, 4/m, 5/m, 8/f	Fever, respiratory symptoms (2 patients), status epilepticus	CSF: Mp PCR pos.	Azi and steroid (all patients), IVIG (3 patients)	Encephalitis	Recovered	[45]
14/f, 7/m, 5/m, 11/f	Respiratory symptoms (2 patients), confusion, gait changes, seizures	Serum IgM/IgG pos.	Azi and IVIG (3 patients), steroid	Encephalitis	Recovered	[46]
9/m	Fever, vomiting, different neurological symptoms	Serum IgM/IgG pos.	Steroid	Encephalitis	Recovered	[47]
13/m	Extremity weakness, neck pain	CSF: IgM/IgG pos.	Doxycycline, steroid, plasmapheresis	Transverse myelitis	Minimal hand and face weakness	[48]
12/f	Bilateral vision loss	PA pos.	Steroid	Optic neuritis	Recovered	[49]

31/m, 33/f, 40/m, 14/f	Fever, headache, different neurological problems	Serum IgM/IgG pos.	Azi (all patients), steroid (1 patient)	MERS	Recovered	[50]
61/m	Different neurological symptoms	Serum IgM/IgG pos.	IVIG	Encephalitis	Recovered	[51]
10/f	Headache, vomiting	Serum IgM pos.	Symptomatic	Encephalopathy	Recovered	[52]
7/m	Fever, cough	Serum IgM pos., pleural effusion: Mp PCR pos.	Azi, steroid, IVIG, heparin	Cerebral infarction	Largely recovered	[53]
21/f	Fever, respiratory symptoms, confusion	Serum IgM pos., respiratory sample: Mp PCR pos.	Azi, steroid, plasmapheresis	ADEM	Unknown	[54]
39/m	Fever, headache	Serum IgM/IgG pos.	Azi	Ischemic stroke, meningoencephalitis	Recovered	[55]
7/f, 20/f	Loss of vision, ocular pain	Serum IgM pos.	Clarithromycin, steroid	Optic neuritis	Recovered	[56]

6-65/ 41.2% m (17 patients)	Different neurological dysfunctions	All patients: PA pos.	15/17 cases: antimicrobial therapy (not specified), steroids (14 patients), IVIG (4 patients)	Encephalitis (7 patients), meningoencephalitis (3 pat.), encephalopathy (3 pat.), meningitis, myelitis, ADEM, BSN (1 pat. each)	13/17 cases recovered	[57]
39/f	Bilateral eye pain	Serum IgM pos.	Clarithromycin, steroid, plasmapheresis	Optic neuritis	Visual field deficits	[58]
8/f	Fever, cough, vomiting, different neurological symptoms	Serum IgM pos.	Azi	Encephalitis	Recovered	[59]
6/m	Visual impairment, headache	Serum IgM/IgG pos.	Azi, steroid, heparin	Ischemic stroke	Largely recovered	[60]
4/m	Fever, cough, different neurological dysfunctions	Serum IgM pos.	Clarithromycin, IVIG	Encephalitis	Recovered	[61]
37/f	Cough, fever, different neurological symptoms	Serum IgM/IgG pos., serum/CSF IgM ratio: 3.8	Clarithromycin, moxifloxacin, tetracycline, steroid	Encephalitis, encephalomyelitis	Incompletely recovered	[62]

14/m, 8/f	Fever, cough, different neurological symptoms	IgM pos.	Both patients: minocycline, steroid (one patient)	MERS	Recovered	[63]
9/m, 12/m	Fever, vomiting, headache, cough, rash	Serum IgM pos.	Azi	MERS	Recovered	[64]
4-14/70% m (10 patients)	Different neurological dysfunctions	Sputum/pharynx: Mp PCR pos., serum IgM pos.	Macrolides (7 patients), steroid (6 patients), IVIG (one patient)	Encephalitis (4 patients), meningitis (3 pat.), meningoencephalitis (2 pat.), transverse myelitis (1 pat.)	Recovered (5 patients), 4 patients with different difficulties	[65]
4/m, 12/f, 9/m, 7/f, 4/f	Fever, headache, ataxia	Serum IgM pos.	Azi (2 patients), doxycycline (3 patients), steroids (all patients), IVIG (3 patients)	Cerebellitis	Recovered (4 patients), one patient with different deficits	[66]
8/m	Fever, cough, pneumonia, neurological symptoms	PA pos., nasopharynx: Mp PCR pos.	Azi, steroid, IVIG	Cerebral infarction	Strong deficit of vision	[67]
5/f	Hemiparesis, facial palsy, pneumonia	Serum IgM pos.	Clarithromycin, steroid	Cerebral infarction	Mild neurological deficits	[68]

4-9/all m (n=7)	Fever, respiratory symptoms	Serum IgM/IgG pos. (71.4%), pharynx: Mp PCR pos. (85.7%), CSF: IgM/IgG pos. in one patient	Clarithromycin (3 patients), doxycycline (2 pat.), quinolones (1 pat.), steroids in 3 pat., IVIG in 2 pat.	Encephalitis	Recovered (4 patients), mild neurological deficits (3 pat.)	[69]
45/f	Fever, headache, paresthesia, loss of vision	Serum IgM/IgG pos.	Steroid	Meningoencephalitis, optic neuritis	Loss of vision, sensory symptoms	[70]

m - male, f - female, pos. - positive, IVIG - intravenous immunoglobulin, azi - azithromycin, Mp - *Mycoplasma pneumoniae*, CSF - cerebrospinal fluid, BAL - bronchoalveolar fluid, PA - particle agglutination test, ADEM - acute disseminated encephalomyelitis, MERS - mild encephalopathy with reversible spinal lesion, BSN - bilateral striatal necrosis.

[a] - only antibiotics effective to mycoplasmas

References

1. Hafez W, Hassan S, Sinha S, Elbaghdady MT, Alhanafy MMA, Mohammed W, Elshekh Ali H, Krishnareddy K. Immune-mediated acute cerebellitis in a patient with *Mycoplasma pneumoniae* infection: A case report and brief review. Radiol Case Rep. **2025**; 20(9):4266-70.
2. Zhao J, Jia N, Zhao J, Wang J, Zhu L. Acute bilateral optic/chiasm neuritis with longitudinally extensive transverse myelitis and positive anti-ganglioside antibodies following *Mycoplasma pneumoniae* pneumonia: a case report and literature review. Neurol Sci. **2025**; 46(9):4269-78.
3. Dhanawade SS, Ghatage PS. Faciobrachial stroke as the initial presentation of Moyamoya in a pediatric patient precipitated by *Mycoplasma pneumoniae* pneumonia: a case report and comprehensive review of the literature. Cureus. **2025**; 17(4):e81979.
4. Horton M, Nguyen ML, Mateus Twitchell M. Atypical cause of autoimmune encephalitis: the role of *Mycoplasma pneumoniae* in status epilepticus. Cureus. **2025**; 17(5):e84689.
5. Meng N, Fan Y, Yin L, Qiao L, Huang L. Severe pneumonia caused by *Mycoplasma pneumoniae* leading to cerebellar infarction with GFAP immune-mediated encephalitis. Indian J Pediatr. **2025**; 92(4):454.
6. Li M, Han Z, Li J, Wang Q, Lv Z. CARP VIII antibody-related autoimmune cerebellar ataxia in a child after *Mycoplasma pneumoniae* infection: a case report. Front Immunol. **2025**; 15:1480212.
7. Madzar D, Nickel FT, Rothhammer V, Goelitz P, Geißdörfer W, Dumke R, Lang R. Meningitis and intracranial abscess due to *Mycoplasma pneumoniae* in a B cell-depleted patient with multiple sclerosis. Eur J Clin Microbiol Infect Dis. **2024**; 43(11):2227-31.
8. Liu YR, Zeng XD, Xiong Y. Myelin oligodendrocyte glycoprotein (MOG) antibody-associated encephalitis induced by *Mycoplasma pneumoniae* infections. Ital J Pediatr. **2024**; 50(1):195.
9. Ye Z, Chen Y, Tian X. Case report: a co-occurring case of severe *Mycoplasma pneumoniae* pneumonia and Anti-IgLON5 antibody-associated encephalitis in a pediatric patient. Front Med (Lausanne). **2024**; 11:1393540.

10. Lacampagne T, Guillotin V, Peuchant O, Belloir A, Sibon I, Bebear C, Cazanave C. Current *Mycoplasma pneumoniae* outbreak: don't forget its role in Bickerstaff encephalitis. Diagn Microbiol Infect Dis. **2024**; 110(1):116418.
11. Xue Y, Yu Z, Cheng M, Li X, Jiang L, Han W. Concomitant central and peripheral nervous system involvement associated with *Mycoplasma pneumoniae* infection in pediatric patients: two case reports and literature review. Pediatr Infect Dis J. **2024**; 43(9):e318-e321.
12. Papantoniou M, Tsatinas K-, Gryllia M. Parainfectious Brown-Sequard syndrome associated with *Mycoplasma pneumoniae* in an adult patient: a case report. Spinal Cord Ser Cases. **2024**; 10(1):13.
13. Jin X, Zuo X. Left middle cerebral artery occlusion associated with mycoplasma pneumonia in a child: a case report. Neuro Endocrinol Lett. **2023**; 44(2):63-7.
14. Fan G, Guo Y, Tang F, Chen M, Liao S, Wang J. Determining the clinical characteristics, treatment strategies, and prognostic factors for *Mycoplasma pneumoniae* encephalitis in children: a multicenter study in China. J Clin Neurol. **2023**; 19(4):402-9.
15. Kammoun S, Rekik M, Maaloul K, Ben Amor S, Trigui A. A rare ocular manifestation of *Mycoplasma pneumoniae* infection. Tunis Med. **2023**; 101(11):855-7.
16. Gayoso-Cantero D, Sarro-Fuentes C, Baron-Rubio M, Losa-García JE. Anisocoria and optic neuritis associated with *Mycoplasma pneumoniae* infection. Enferm Infecc Microbiol Clin (Engl Ed). **2023**; 41(5):312-4.
17. Yoshimoto K, Matsubara M, Kobayashi T, Nishio K. A case of mycoplasma infection with an atypical presentation of abducens nerve palsy, erythema multiforme and polyarthritis without respiratory manifestations. Medicina (Kaunas). **2023**; 60(1):36.
18. Huang X, Guo R, Li C, Long X, Yang T, Hou X, Wei X, Ou M. A case of anti-myelin oligodendrocyte glycoprotein (MOG)-immunoglobulin G (IgG) associated disorder (MOGAD) with clinical manifestations of acute disseminated encephalomyelitis: secondary to *Mycoplasma pneumoniae* infection. Heliyon. **2023**; 9(2):e13470.
19. Zeng XD, Chen H, Hu WG. Myelin Oligodendrocyte Glycoprotein (MOG) antibody-associated meningoencephalitis due to *Mycoplasma pneumoniae* infection. Neurol Res. **2023**; 45(2):124-6.

20. Lu KH, Wu TC, Yeh PS. Cytotoxic lesions beyond the corpus callosum following acute meningoencephalitis and *Mycoplasma pneumoniae* infection: a case report and literature review. Case Rep Neurol. **2023**; 15(1):113-9.

21. Yadava SK, Adhikari S, Ojha N, Zaidi SH, Hanish J, Fazili T. Stevens-Johnson syndrome and stroke related to mycoplasma. J Investig Med High Impact Case Rep. **2022**; 10:23247096211067975.

22. Gonzalez-Rodriguez B, Gonzalez-Rodriguez M, Bejarano Ramírez N, Redondo Calvo FJ. Optic neuritis as sign presentation of acute disseminated encephalomyelitis following *Mycoplasma pneumoniae* infection. Rev Esp Quimioter. **2022**; 35(2):223-4.

23. Been Sayeed SKJ, Moniruzzaman M, Mahmud R, Rashid MB, Chandra Das S. Acute hemorrhagic leucoencephalitis (AHLE): a rare CNS presentation of *Mycoplasma pneumoniae*. Cureus. **2022**; 14(10):e30921.

24. Khan A, Haq AU, Hamid H, Fatima T, Adnan S. Rhombencephalitis possibly caused by *Mycoplasma pneumoniae*. J Ayub Med Coll Abbottabad. **2022**; 34(3):566-8.

25. Khunkhun S, Aggarwal K, Iqbal H, Satyadev N, Mann K, Ruxmohan S, Perez G, Tamton H. A rare presentation of a complex mixed autoimmune encephalitis diagnosis: a case report and literature review. Cureus. **2022**; 14(9):e29607.

26. Monte G, Pro S, Ursitti F, Ferilli MAN, Moavero R, Papetti L, Sforza G, Bracaglia G, Vigevano F, Palma P, Valeriani M. Case report: a pediatric case of Bickerstaff brainstem encephalitis after COVID-19 vaccination and *Mycoplasma pneumoniae* infection: looking for the culprit. Front Immunol. **2022**; 13:987968.

27. Talukder NT, Feezel A, Lankford JE. Mild encephalitis/encephalopathy with a reversible splenial lesion associated with systemic *Mycoplasma pneumoniae* infection in North America: a case report. J Med Case Rep. **2022**; 16(1):74.

28. Rodriguez-Montolio J, Ballesta-Martinez S, Martin-Aleman Y, Munoz-Farjas E. Acute disseminated encephalomyelitis after *Mycoplasma pneumoniae* infection: unfavourable clinical course, excellent recovery. Neurologia (Engl Ed). **2022**; 37(4):313-5.

29. Woo MH, Shin JW. Acute brainstem encephalitis associated with *Mycoplasma pneumoniae* in an adult: a case report. Encephalitis. **2021**; 1(4):120-3.

30. Wang Y, Xiao Y, Deng X, Xu N, Chen Z. Cardiac thrombus and stroke in a child with *Mycoplasma pneumoniae* pneumonia: a case report. Medicine (Baltimore). **2021**; 100(5):e24297.

31. Oberoi M, Kulkarni R, Oliver T. An unusual case of myocarditis, left ventricular thrombus, and embolic stroke caused by *Mycoplasma pneumoniae*. Cureus. **2021**; 13(3):e14170.

32. Zhang Y, Shi Q. A wide range of high signal intensities on brain image in adult *Mycoplasma pneumoniae*-associated mild encephalitis/encephalopathy with a reversible splenial lesion. Neurol India. **2021**; 69(4):1112-3.

33. Akbar A, Ahmad S. Atypical case of mild encephalopathy/encephalitis with reversible splenial lesion of the corpus callosum (MERS) associated with *Mycoplasma pneumoniae* infection in a paediatric patient. BMJ Case Rep. **2021**; 14(8):e242791.

34. Ding G, Song D, Vinturache A, Gu H, Zhang Y. Cerebral infarction associated with *Mycoplasma pneumoniae* infection in a child. Pediatr Int. **2021**; 63(8):978-80.

35. Kc O, Dahal PH, Koirala M, NtemMensah AD. Rhabdomyolysis and neurological manifestation with progressive weakness in a young adult: a rare extrapulmonary presentation of *Mycoplasma pneumoniae*. Cureus. **2021**; 13(12):e20552.

36. He CB, Lee JR, Kahana M. *Mycoplasma pneumoniae* associated acute transverse myelitis: an atypical clinical presentation in an adolescent child. Cureus. **2021**; 13(8):e17259.

37. Bonagiri P, Park D, Ingebritsen J, Christie LJ. Seropositive anti-MOG antibody-associated acute disseminated encephalomyelitis (ADEM): a sequelae of *Mycoplasma pneumoniae* infection. BMJ Case Rep. **2020**; 13(5):e234565.

38. Ahmed AOE, Babikir MMI, Khojali AEM, Arachchige SNM, Abdirahman AM, Mohamed MFH. Central nervous system vasculitis as a rare presentation of *Mycoplasma pneumoniae*: a case report. Case Rep Neurol. **2020**; 12(3):402-9.

39. Sadohara M, Arai T, Matsuura K. Clinically mild encephalitis/encephalopathy with reversible splenial lesion (MERS) associated with *Mycoplasma pneumoniae* pneumonia: an adult case and review of the literature. Clin Case Rep. **2020**; 8(12):2955-61.
40. Akkus A, Torun EG, Yazici MU, Azapagasi E, Sahap SK, Danis A, Aksoy A. Severe mycoplasma encephalitis in a child: the corticosteroid treatment. Klin Padiatr. **2020**; 232(4):219-21.
41. Molero-Senosiain M, Domingo-Gordo B, Fernandez Cabrera C, Hernandez-García E, Gomez de Liano R. Neuro-ophthalmological manifestations as complication of an infection with *Mycoplasma pneumoniae* and subsequent development of disseminated acute encephalitis. Arch Soc Esp Oftalmol (Engl Ed). **2020**; 95(5):254-8.
42. Coriolani G, Ferranti S, Squarci G, Grosso S. A case of Bickerstaff encephalitis associated with *Mycoplasma pneumoniae* infection. Neurol Sci. **2020**; 41(6):1605-6.
43. Yildirim M, Gocmen R, Konuskan B, Parlak S, Yalnizoglu D, Anlar B. Acute cerebellitis or postinfectious cerebellar ataxia? Clinical and imaging features in acute cerebellitis. J Child Neurol. **2020**; 35(6):380-8.
44. Xia C, Chen HS. Anti-GM2 antibodies in *Mycoplasma pneumoniae*-associated acute encephalomyelitis. Can J Neurol Sci. **2020**; 47(2):258-60.
45. Feng S, Chen JX, Zheng P, Zhang JZ, Gao ZJ, Mao YY, Ji XN, Chen SH, Sun HM, Chen Q. Status epilepticus associated with *Mycoplasma pneumoniae* encephalitis in children: good prognosis following early diagnosis and treatment. Chin Med J (Engl). **2019**; 132(12):1494-6.
46. Daba M, Kang PB, Sladky J, Bidari SS, Lawrence RM, Ghosh S. Intravenous immunoglobulin as a therapeutic option for *Mycoplasma pneumoniae* encephalitis. J Child Neurol. **2019**; 34(11):687-91.
47. Smolders J, Jacobs BC, Tio-Gillen AP, Nijhuis F, Verrips A. *Mycoplasma pneumoniae* and antibodies against galactocerebroside in a 9-year-old boy with encephalitis. Neuropediatrics. **2019**; 50(1):54-6.
48. Salloum S, Goenka A, Ey E. *Mycoplasma pneumoniae* associated transverse myelitis presenting as asymmetric flaccid paralysis. Clin Pract. **2019**; 9(3):1142.

49. Matsunaga M, Kodama Y, Maruyama S, Miyazono A, Seki S, Tanabe T, Yoshimura M, Nishi J, Kawano Y. Guillain-Barré syndrome and optic neuritis after *Mycoplasma pneumoniae* infection. Brain Dev. **2018**; 40(5):439-42.

50. Dong X, Cong S. Reversible splenial lesion syndrome associated with acute *Mycoplasma pneumoniae*-associated encephalitis: a report of four cases and literature review. Exp Ther Med. **2018**; 16(3):2152-9.

51. Puma A, Benoit J, Sacconi S, Uncini A. Miller Fisher syndrome, Bickerstaff brainstem encephalitis and Guillain-Barré syndrome overlap with persistent non-demyelinating conduction blocks: a case report. BMC Neurol. **2018**; 18(1):101.

52. Ramgopal A, Thavamani A, Ghori A. Association between posterior reversible encephalopathy syndrome and *Mycoplasma pneumoniae* infection. J Pediatr Neurosci. **2018**; 13(1):109-11.

53. Jin X, Zou Y, Zhai J, Liu J, Huang B. Refractory *Mycoplasma pneumoniae* pneumonia with concomitant acute cerebral infarction in a child: a case report and literature review. Medicine (Baltimore). **2018**; 97(13):e0103.

54. Laila A, El-Lababidi RM, Hisham M, Mooty M. A case of acute disseminated encephalomyelitis following *Mycoplasma pneumoniae* infection. IDCases. **2018**; 12:41-3.

55. Choi SY, Choi YJ, Choi JH, Choi KD. Isolated optic neuritis associated with *Mycoplasma pneumoniae* infection: report of two cases and literature review. Neurol Sci. **2017**; 38(7):1323-7.

56. Sarathchandran P, Al Madani A, Alboudi AM, Inshasi J. *Mycoplasma pneumoniae* infection presenting as stroke and meningoencephalitis with aortic and subclavian aneurysms without pulmonary involvement. BMJ Case Rep. **2018**; 2018:bcr2017221831.

57. Kuwahara M, Samukawa M, Ikeda T, Morikawa M, Ueno R, Hamada Y, Kusunoki S. Characterization of the neurological diseases associated with *Mycoplasma pneumoniae* infection and anti-glycolipid antibodies. J Neurol. **2017**; 264(3):467-75.

58. Baheerathan A, Ross Russell A, Bremner F, Farmer SF. A rare case of bilateral optic neuritis and Guillain-Barré syndrome post *Mycoplasma pneumoniae* infection. Neuroophthalmology. **2016**; 41(1):41-7.
59. Lin YL, Hung KL, Lo CW. *Mycoplasma pneumoniae*- associated encephalitis complicated by cerebral salt wasting syndrome. Clin Case Rep. **2017**; 5(11):1830-3.
60. Garcia Tirado A, Jimenez-Rolando B, Noval S, Martinez Bermejo A. Cortical blindness in a child secondary to *Mycoplasma pneumoniae* infection. J Stroke Cerebrovasc Dis. **2017**; 26(1):e12-e13.
61. Saker A, Athman S, Aldosari M, Frayha H. Encephalopathy and axonal neuropathy associated with *Mycoplasma pneumoniae* infection: response to intravenous immunoglobulin therapy. Child Neurol Open. **2016**; 3:2329048X16632140.
62. Horvath T, Fischer U, Müller L, Ott S, Bassetti CL, Wiest R, Sendi P, Schefold JC. Mycoplasma-induced minimally conscious state. Springerplus. **2016**; 5:143.
63. Ueda N, Minami S, Akimoto M. *Mycoplasma pneumoniae*-associated mild encephalitis/encephalopathy with a reversible splenial lesion: report of two pediatric cases and a comprehensive literature review. BMC Infect Dis. **2016**; 16(1):671.
64. Yuan ZF, Shen J, Mao SS, Yu YL, Xu L, Jiang PF, Gao F, ZZ. Clinically mild encephalitis/encephalopathy with a reversible splenial lesion associated with *Mycoplasma pneumoniae* infection. BMC Infect Dis. **2016**; 16:230.
65. Kammer J, Ziesing S, Davila LA, Bültmann E, Illsinger S, Das AM, Haffner D, Hartmann H. Neurological manifestations of *Mycoplasma pneumoniae* infection in hospitalized children and their long-term follow-up. Neuropediatrics. **2016**; 47(5):308-17.
66. Kornreich L, Shkalim-Zemer V, Levinsky Y, Abdallah W, Ganelin-Cohen E, Straussberg R. Acute cerebellitis in children: a many-faceted disease. J Child Neurol. **2016**; 31(8):991-7.
67. Bao Y, Li X, Wang K, Zhao C, Ji X, Jiang M. Central retinal artery occlusion and cerebral infarction associated with *Mycoplasma pneumoniae* infection in children. BMC Pediatr. **2016**; 16(1):210.

68. Kang B, Kim DH, Hong YJ, Son BK, Lim MK, Choe YH, Kwon YS. Complete occlusion of the right middle cerebral artery associated with *Mycoplasma pneumoniae* pneumonia. Korean J Pediatr. **2016**; 59(3):149-52.

69. Meyer Sauteur PM, Moeller A, Relly C, Berger C, Plecko B, Nadal D; Swiss Pediatric Surveillance Unit (SPSU). Swiss national prospective surveillance of paediatric *Mycoplasma pneumoniae*-associated encephalitis. Swiss Med Wkly. **2016**; 146:w14222.

70. Benedetti L, Franciotta D, Beronio A, Delucchi S, Capellini C, Del Sette M. Meningoencephalitis-like onset of post-infectious AQP4-IgG-positive optic neuritis complicated by GM1-IgG-positive acute polyneuropathy. Mult Scler. **2015**; 21(2):246-8.
